# Latissimus dorsi flap: a comprehensive systematic review of traditional and novel applications

**DOI:** 10.3389/fsurg.2026.1752461

**Published:** 2026-03-13

**Authors:** María García-García, Belén Andresen-Lorca, Alessandro Thione, Pedro Alvedro-Ruiz, Arantxa Blasco-Serra, Eva M. González-Soler, Alfonso A. Valverde-Navarro

**Affiliations:** 1 Plastic Surgery and Burns Department, La Fe Hospital, Valencia, Spain; 2 Human Anatomy and Embriology Department, Universitat de Valencia, Valencia, Spain

**Keywords:** latissimus dorsi, LD flap, microsurgery, plastic surgery, reconstructive surgery

## Abstract

**Background:**

The latissimus dorsi (LD) flap is among the most adaptable and extensively employed techniques in reconstructive surgery, providing a dependable solution for addressing a wide range of defects. Although traditionally employed for soft-tissue coverage, its indications have progressively expanded to include complex and functional reconstructions. The aim of this review was to provide a comprehensive, indication-based overview of both traditional and emerging applications of the LD flap reported in the literature.

**Method:**

A systematic review was conducted in accordance with PRISMA guidelines, analysing published applications of the LD flap in reconstructive surgery. An initial search was performed up to July 30, 2024, and subsequently updated during manuscript revision to include studies published through January 10, 2026. Two independent reviewers examined the selected articles, individually extracting the relevant data, which was later combined and contrasted. Given the descriptive aim of the review, no statistical analysis was performed.

**Results:**

A total of 188 full-text articles were included. Based on anatomical location, indications were categorized into breast, head and neck, thorax and back, abdomen and pelvis, and upper and lower extremity reconstruction. Both coverage and functional applications were identified. Functional reconstructions included innervated and non-innervated LD flaps used for facial reanimation, limb motor restoration, urologic reconstruction (including phalloplasty and detrusor myoplasty), diaphragmatic reconstruction, and other dynamic applications. Pedicled LD flaps were predominantly reported for anatomically adjacent defects, whereas free LD flaps were more commonly used for distant or complex reconstructions.

**Conclusions:**

The latissimus dorsi flap can be configured in multiple forms to address defects across nearly all anatomical regions. Contemporary literature demonstrates a clear evolution toward functional and indication-driven applications, supported by advances in microsurgical techniques and muscle reinnervation. This descriptive synthesis provides a structured overview of reported indications and may assist surgeons in selecting reconstructive strategies based on anatomical and functional objectives.

## Introduction

The latissimus dorsi (LD) flap is one of the most versatile and widely utilized options in reconstructive surgery, offering a reliable solution for virtually any type of defect. It employs the latissimus dorsi muscle, a broad, flat muscle located in the posterior thorax, which is primarily supplied by the thoracodorsal artery and vein, branches of the subscapular system, and innervated by the thoracodorsal nerve (1).

First introduced in 1906, the LD flap was initially used for breast reconstruction following mastectomy (2). Over time, its applications have expanded significantly, making it a cornerstone in reconstructive surgery. The flap provides a well-vascularized, pliable, and durable tissue, which can reconstruct defects in multiple anatomical regions, including the head and neck, chest wall, upper and lower extremities, back and pelvis. Additionally, the LD flap has been proven valuable in both functional and aesthetic reconstruction, with modifications such as innervated flaps for restoring muscle function and perforator-based techniques for minimizing donor site morbidity.

Despite its widespread use, the indications, advantages, and limitations of the LD flap continue to evolve and grow. While some surgeons favor it due to its reliable vascularity and large surface area, concerns related to donor site morbidity, muscle atrophy, and functional impairment (3–5) have led to ongoing refinements in surgical techniques [such as muscle-sparing flaps (6–8)]. Innovations such as robot-assisted (9–12) and endoscopic (6, 13, 14) harvest, fat grafting (15–17), neuromuscular reinnervation, and the use of chimeric or hybrid flaps have further expanded its reconstructive potential.

This systematic review aims to provide a comprehensive analysis of all applications of the latissimus dorsi flap in reconstructive surgery. By compiling its described uses across different anatomical regions and surgical contexts, we seek to highlight the versatility, benefits, and evolving role of this flap in modern microsurgical reconstruction. Through this work, we aspire to offer a comprehensive guide for both novice and experienced surgeons, that will serve as a reference to easily review all possible applications of this versatile flap in reconstructive surgery. Organized by anatomical regions, this guide should assist and inspire surgeons when in search of reconstructive options facing a specific defect.

## Material and methods

### Search strategy

We conducted a systematic review in accordance with the Preferred Reporting Items for Systematic Reviews and Meta-Analyses (PRISMA) and AMSTAR 2 (18) guidelines on the published uses of the latissimus dorsi flap in reconstructive surgery.

One electronic database (*Medline*) was queried from June 4, 2024 to July 30, 2024 using the following research strategy: [superficial back muscles/surgery(MeSH Terms)] AND ([surgery, plastic(MeSH Terms)] OR [Plastic Surgery Procedures(MeSH Terms)]). In response to the peer-review process, the literature search was subsequently updated to include newly published studies, with the final search performed on January 10, 2026. Relevant additional articles identified through this update were incorporated into the final analysis and tables.

### Inclusion and exclusion criteria

We included all articles meeting the following criteria: articles published in English, Spanish or French (languages spoken by both reviewers), with full-text freely available or retrievable via our institution's library (Hospital La Fe), published in indexed journals, on common and original indications of the latissimus dorsi flap in reconstructive surgery.

We excluded publications in conferences or congresses, letters to the editor, press releases, editorials, comments, reviews and cohort studies. In such a way, only case-series were collected, as the purpose of our study was to compile only the reported indications the flap had been used for and not comparing or contrasting it with other reconstructive methods.

### Data extraction and evaluation

Both reviewers independently revised the selected articles, individually extracting the relevant data, which was later combined and contrasted. The following data was collected: author, defect location and indication, flap type (pedicled or free), recipient vessels and presence or absence of reinnervation.

The methodological quality and risk of bias of the included case reports and case series were assessed using the Joanna Briggs Institute (JBI) Critical Appraisal Checklist for Case Reports. This checklist evaluates key aspects such as the clarity of the clinical presentation, the appropriateness of the interventions and outcomes reported, follow-up duration, and ethical considerations. Each included study was appraised independently by two reviewers, with disagreements resolved through discussion or consultation with a third reviewer.

Given the purely descriptive aim of this review, no statistical analysis was performed. Instead, the published indications of the latissimus dorsi flap were systematically compiled and organized to allow readers to appreciate the breadth and versatility of its reported applications.

## Results

**Figure 1:**
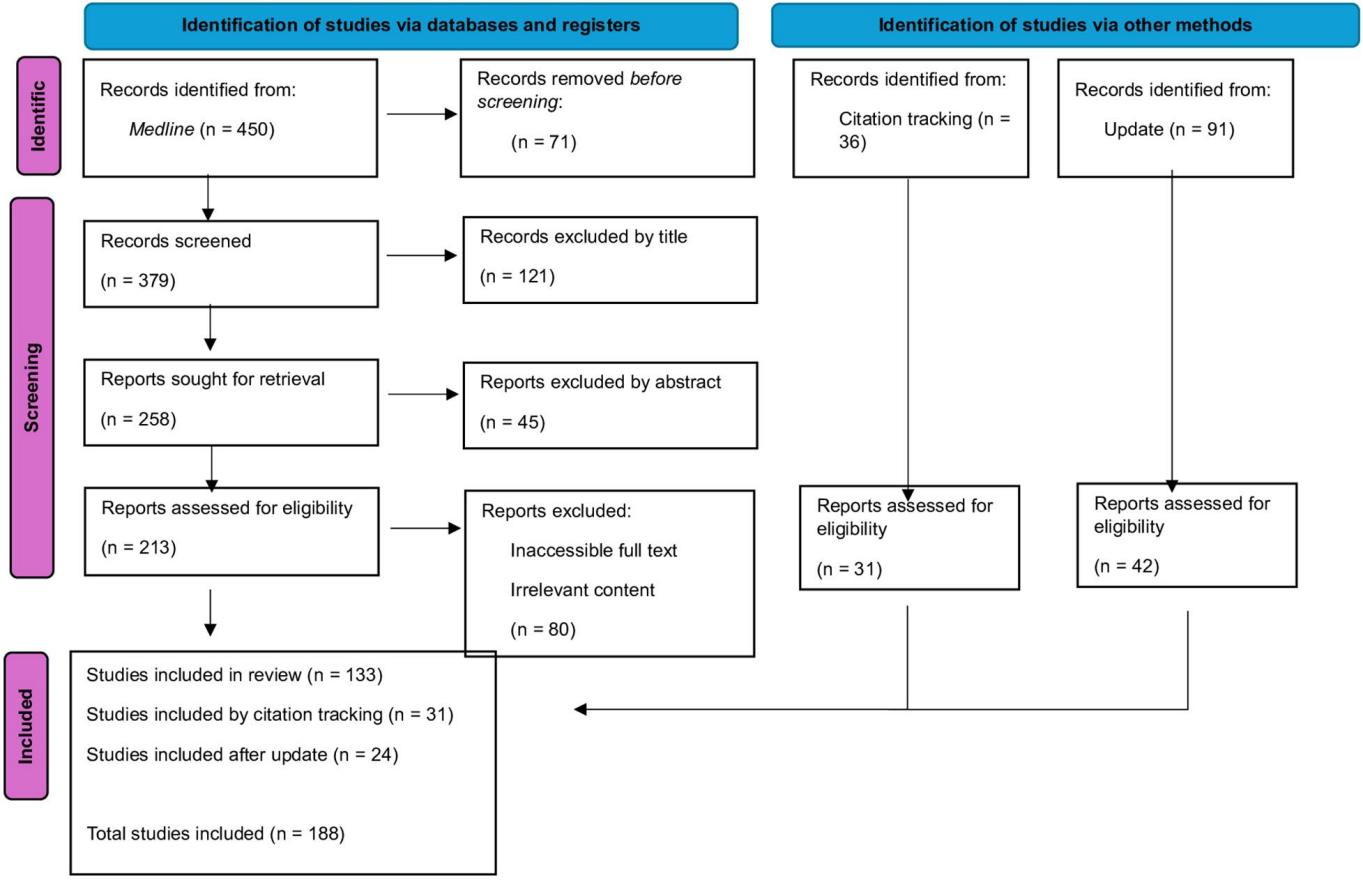
PRISMA flow diagram. This figure shows the number of records identified, screened, and included in the systematic review, along with reasons for exclusion at each stage, following PRISMA 2020 guidelines.

Our search yielded 450 articles. Initial screening reduced the sample to 379 publications. After reading all titles and abstracts and applying our exclusion criteria, only 213 articles were selected. Finally, following full-text reading, 133 articles were included in our review. Citation tracking identified an additional 31 relevant studies, resulting in 164 articles included from the initial search. During the revision of the manuscript, the literature search was updated to include newly published studies. From this update, 24 additional articles deemed most relevant to the scope of the review were incorporated into the final analysis and tables, bringing the total number of included full-text articles to 188. Figure 1 represents the PRISMA flow diagram. The results of the quality assessment for each included study are summarized in the Supplementary Material (Supplementary Table S1).

Table 1 (with results sorted by publication date) summarizes the characteristics of the 188 selected articles, which included 76 case reports and 112 case series. Although most studies consisted of single-case reports or small series, several large retrospective series were also identified, reflecting the predominantly descriptive nature of the available evidence. All studies had been conducted in the last 40 years, with the oldest dating back to 1983 and the most recent to 2025.

**Table 1:** Selected articles’ characteristics.

Author	Study design	Year of publication	Sample (*n*)
Bianchi et al. (19)	Case series	1983	3
Sabatier et al. (20)	Case series	1992	63
Zimmermann et al. (21)	Case report	1993	1
Yap et al. (22)	Case report	1994	1
Papadopoulos et al. (23)	Case series	1994	12
Allen et al. (24)	Case series	1994	12
Bootz et al. (25)	Case series	1995	7
Wallace and Roden (26)	Case series	1995	5
Whetzel et al. (27)	Case report	1997	1
Schultes et al. (28)	Case report	1998	1
Park and Koh (29)	Case report	1998	1
Bedini et al. (30)	Case series	2000	9
Ihara et al. (31)	Case series	2000	12
Dixon et al. (32)	Case series	2002	25
Meiners (33)	Case report	2003	1
De Smet et al. (34)	Case report	2004	1
Germann et al. (35)	Case report	2006	1
McConkey et al. (36)	Case report	2006	1
Djordjevic et al. (37)	Case series	2006	30
Perovic et al. (38)	Case series	2007	16
Hierner et al. (39)	Case series	2009	14
Gakis et al. (40)	Case series	2011	11
Ozer et al. (41)	Case report	2011	1
Phan et al. (42)	Case series	2011	8
He et al. (43)	Case series	2012	11
Kim et al. (44)	Case series	2012	20
Kim et al. (45)	Case report	2012	1
Gangurde et al. (46)	Case series	2012	3
Qu et al. (47)	Case series	2012	6
Nazerani et al. (48)	Case series	2012	7
Hwang et al. (49)	Case report	2012	1
Venkatramani et al. (50)	Case series	2013	19
Biglioli et al. (51)	Case series	2013	17
Karle et al. (52)	Case series	2013	3
Tsai et al. (53)	Case report	2013	1
Takushima et al. (54)	Case series	2013	351
Patel et al. (55)	Case report	2014	1
Trimaille et al. (56)	Case report	2014	1
Vibhakar et al. (57)	Case report	2014	1
Endara et al. (58)	Case report	2014	1
Fujioka et al. (59)	Case report	2014	1
Muramatsu et al. (60)	Case series	2014	4
Hornez et al. (61)	Case report	2014	1
Kim et al. (62)	Case report	2014	7
Santanelli di Pompeo et al. (63)	Case series	2014	23
Hillerup et al. (64)	Case series	2014	15
Wilkman et al. (65)	Case series	2014	10
Wormald et al. (66)	Case series	2014	15
Horta et al. (67)	Case report	2015	1
Okazaki et al. (68)	Case series	2015	4
Gilleard et al. (69)	Case report	2015	1
Tuncer et al. (70)	Case report	2015	1
Cigna et al. (71)	Case report	2015	1
Højvig y Bonde (72)	Case series	2015	135
Jain et al. (73)	Case report	2015	1
Kaur et al. (74)	Case series	2015	19
Kesiktas et al. (75)	Case series	2015	12
Longo et al. (76)	Case series	2015	5
Miyamoto et al. (77)	Case series	2015	5
Singh et al. (78)	Case series	2015	2
Tan et al. (79)	Case series	2015	2
Angrigiani et al. (80)	Case series	2015	45
Bach et al. (81)	Case series	2015	7
Nicoli et al. (82)	Case report	2016	1
Rednam and Rinker (83)	Case report	2016	1
Buchanan et al. (84)	Case report	2016	1
Banys-Paluchowski et al. (85)	Case report	2016	1
He et al. (86)	Case series	2016	12
Ng et al. (87)	Case report	2016	1
Amin et al. (88)	Case report	2016	1
Bodin et al. (89)	Case report	2016	1
Cadenelli et al. (90)	Case report	2016	1
Ozcan Akcal et al. (91)	Case series	2016	2
Correia Anacleto et al. (92)	Case series	2016	100
Gao et al. (93)	Case series	2016	16
Gencel et al. (94)	Case series	2016	6
Hwang et al. (95)	Case series	2016	23
Lü et al. (96)	Case series	2016	15
Mutlu et al. (97)	Case report	2016	1
Miyamoto et al. (98)	Case series	2016	2
Homma et al. (99)	Case report	2017	1
Azab and Alsabbahi (100)	Case series	2017	13
Kamochi et al. (101)	Case series	2017	2
Morice et al. (102)	Case series	2017	1
Park et al. (103)	Case series	2017	4
Tenekeci et al. (104)	Case report	2017	1
Takahashi et al. (105)	Case report	2017	1
Bodin et al. (106)	Case series	2017	9
Ortmaier et al. (107)	Case series	2017	116
Cook et al. (7)	Case series	2017	126
De Runz et al. (108)	Case series	2017	122
DeLong et al. (109)	Case series	2017	2,304
Yuan et al. (110)	Case series	2017	25
Papadakis et al. (111)	Case report	2017	1
Vairinho et al. (112)	Case report	2018	1
Beltrami et al. (113)	Case series	2018	2
Kang et al. (114)	Case series	2018	2
Ju et al. (115)	Case series	2018	7
Marchesi et al. (116)	Case series	2018	12
Lai et al. (117)	Case report	2018	1
Voss et al. (118)	Case series	2019	3
Barrientos et al. (119)	Case report	2019	1
Fried et al. (120)	Case report	2019	1
Wong et al. (121)	Case report	2019	1
Tashiro et al. (122)	Case series	2019	6
Djordjevic et al. (123)	Case series	2019	129
Dutra et al. (124)	Case series	2019	123
Maselli et al. (125)	Case series	2019	7
Mericli et al. (126)	Case series	2019	47
Motono et al. (127)	Case report	2019	1
Boonipat et al. (128)	Case report	2019	1
Hallock (129)	Case report	2019	1
Hamada et al. (130)	Case report	2019	1
Li et al. (131)	Case series	2019	11
Liu et al. (132)	Case series	2019	16
Lohana et al. (133)	Case series	2019	120
Matsumine et al. (134)	Case series	2019	2
Özkan et al. (135)	Case series	2019	12
Scaglioni and Giunta (136)	Case series	2019	5
Khan et al. (137)	Case report	2020	1
Menichini et al. (138)	Case report	2020	1
Mughal et al. (139)	Case series	2020	11
Chim et al. (8)	Case series	2020	42
Dragos et al. (140)	Case report	2020	1
Cha et al. (15)	Case series	2020	45
De Lorenzi et al. (141)	Case series	2020	11
Piat et al. (142)	Case series	2020	72
Sakharpe et al. (143)	Case report	2020	1
Xiao et al. (144)	Case report	2020	1
Song et al. (145)	Case series	2020	2
Bedarida et al. (146)	Case series	2020	8
Ogawa et al. (147)	Case report	2021	1
Ogawa et al. (148)	Case series	2021	3
Münchow et al. (149)	Case report	2021	1
Raymond et al. (150)	Case series	2021	18
Ahmed et al. (17)	Case series	2021	25
Casella et al. (151)	Case series	2021	23
Couto-González et al. (16)	Case series	2021	95
Alshammari et al. (152)	Case report	2021	1
Valenti et al. (153)	Case series	2021	16
Chang et al. (154)	Case series	2021	25
Chang et al. (155)	Case series	2022	10
Brunetti et al. (156)	Case report	2022	1
Fraisse et al. (157)	Case report	2022	1
Zhao et al. (158)	Case report	2022	1
Zhang et al. (159)	Case series	2022	13
Klein et al. (160)	Case report	2022	1
Amendola et al. (161)	Case report	2022	1
He et al. (162)	Case series	2022	72
Miura et al. (163)	Case report	2022	1
Feng et al. (164)	Case series	2022	20
Yoo et al. (165)	Case series	2022	30
Ozaniak et al. (166)	Case report	2022	1
Zheng et al. (167)	Case series	2022	31
Lee et al. (168)	Case series	2022	97
Brambilla et al. (169)	Case series	2022	12
Homsy et al. (170)	Case series	2022	126
Katayama et al. (171)	Case series	2023	3
Alessandri-Bonetti et al. (172)	Case report	2023	1
Kagaya et al. (173)	Case report	2023	1
Brunetti et al. (174)	Case report	2023	1
Chen et al. (9)	Case series	2023	15
Bota et al. (175)	Case report	2023	1
Alonso-Rodriguez Piedra et al. (176)	Case series	2023	10
Adamyan et al. (177)	Case series	2023	592
Lellouch et al. (178)	Case report	2023	1
Anghel et al. (179)	Case report	2023	1
Olvera-Yarza et al. (180)	Case report	2023	1
Yang et al. (181)	Case report	2023	1
Kim et al. (182)	Case report	2023	1
Thione et al. (183)	Case series	2023	2
Jaffar et al. (184)	Case series	2023	26
Sui et al. (185)	Case series	2023	53
La Padula et al. (186)	Case series	2023	68
Marque et al. (187)	Case report	2023	1
D'Alessandro et al. (188)	Case series	2024	176
Banys-Paluchowski et al. (189)	Case series	2024	140
Bassiri Gharb et al. (190)	Case series	2024	10
Fujioka and Koga (191)	Case report	2024	1
Javeed et al. (192)	Case series	2024	184
Trapero et al. (193)	Case report	2024	1
Wu et al. (194)	Case series	2025	65
Nasır (195)	Case series	2025	30
Ahmed et al. (196)	Case series	2025	18
Rodríguez et al. (197)	Case report	2025	1
Mordovskiy et al. (198)	Case series	2025	32
Panzenbeck et al. (199)	Case series	2025	52
Fragomen et al. (200)	Case report	2025	1

Based on anatomical location, the included articles were categorized into six major reconstructive domains: breast reconstruction (39 articles), head and neck reconstruction (34 articles), thorax and back reconstruction (28 articles), abdominal and pelvic reconstruction (6 articles), lower limb reconstruction (23 articles), and upper limb reconstruction (10 articles).

Regarding its reconstructive purpose, 25 articles described functional applications of the innervated latissimus dorsi flap, while 35 articles reported non-innervated flap reconstructions used with a functional purpose. Moreover, 10 of the functional reconstructions mentioned (5 innervated and 7 non-innervated) had a double purpose, involving both defect coverage and functional restoration.

Breast reconstruction represented the most frequently reported indication for the LD flap. The flap was primarily employed for oncologic breast reconstruction following mastectomy or lumpectomy, particularly in patients undergoing radiotherapy and/or chemotherapy. Most reconstructions were performed using pedicled flaps, often combined with implants, tissue expanders, or fat grafting to achieve adequate breast volume in either immediate or delayed settings. Free flaps were less commonly reported in breast reconstruction; when used, the internal mammary vessels were the most frequently selected recipients. Notably, across all reviewed studies, no cases of voluntary or involuntary reinnervation of the LD flap were reported in the context of breast reconstruction. Detailed characteristics of breast reconstruction cases are summarized in Table 2.

**Table 2:** Use of latissimus dorsi for breast reconstruction.

Author	Flap indication	Flap type	Recipient vessels	Reinnervation
Dixon et al. (32)	Breast reconstruction after mastectomy	Pedicled (mini-flap)	–	No
Vibhakar et al. (57)	Breast reconstruction after mastectomy and lymphadenectomy	Pedicled (+ lateral thoracic lymph nodes transfer)	–	No
Endara et al. (58)	Breast reconstruction after mastectomy	Free	Internal mammary	No
Santanelli di Pompeo et al. (63)	Breast reconstruction after mastectomy	Pedicled (+ fat graft)	–	No
Højvig y Bonde (72)	Breast reconstruction after mastectomy	Pedicled	–	No
Kaur et al. (74)	Breast reconstruction after mastectomy	Pedicled	–	No
Correia Anacleto et al. (92)	Breast reconstruction after mastectomy	Pedicled (kite flap)	–	No
Banys-Paluchowski et al. (85)	Thoracic wall reconstruction after male mastectomy	Pedicled	–	No
He et al. (86)	Breast reconstruction in Poland syndrome	Pedicled chimeric (+ thoracodorsal artery perforator flap)	–	No
Cook et al. (7)	Breast reconstruction after mastectomy	Pedicled	–	No
De Runz et al. (108)	Breast reconstruction after mastectomy and RT	Pedicled	–	No
DeLong et al. (109)	Breast reconstruction after mastectomy and RT	Pedicled	–	No
Yuan et al. (110)	Breast reconstruction after partial or total mastectomy	Pedicled (laparoscopic)	–	No
Lai et al. (117)	Breast reconstruction after lumpectomy	Pedicled (laparoscopic)	–	No
Lee et al. (168)	Breast reconstruction after mastectomy	Pedicled (vertical and mini-flap)	–	No
Dutra et al. (124)	Breast reconstruction after mastectomy and RT	Pedicled	–	No
Li et al. (131)	Breast reconstruction after radionecrosis	Pedicled	–	No
Liu et al. (132)	Breast reconstruction in Poland syndrome	Pedicled	–	No
Lohana et al. (133)	Breast reconstruction after mastectomy	Pedicled (bilateral)	–	No
Maselli et al. (125)	Breast reconstruction after mastectomy	Pedicled	–	No
Mericli et al. (126)	Breast reconstruction after lumpectomy	Pedicled	–	No
Motono et al. (127)	Breast and thorax reconstruction after tumoral excision	Pedicled	–	No
Cha et al. (15)	Breast reconstruction after mastectomy	Pedicled (+ fat graft)	–	No
De Lorenzi et al. (141)	Breast reconstruction after mastectomy	Pedicled	–	No
Piat et al. (142)	Breast reconstruction after mastectomy	Pedicled (+ fat graft)	–	No
Sakharpe et al. (143)	Breast reconstruction after mastectomy	Reverse pedicled based on intercostal arteries	–	No
Song et al. (145)	Breast reconstruction after mastectomy	Pedicled (bilobed)	–	No
Ahmed et al. (17)	Breast reconstruction after mastectomy	Pedicled (+ fat graft)	–	No
Casella et al. (151)	Breast reconstruction after mastectomy	Pedicled (kite flap)	–	No
Couto-González et al. (16)	Breast reconstruction after mastectomy	Pedicled (+ fat graft)	–	No
Feng et al. (164)	Breast reconstruction after mastectomy	Pedicled (laparoscopic)	–	No
Zheng et al. (167)	Breast reconstruction after mastectomy	Pedicled (vertical)	–	No
Chen et al. (9)	Breast reconstruction after mastectomy	Pedicled (Da Vinci)	–	No
D'Alessandro et al. (188)	Breast reconstruction after mastectomy	Pedicled	–	No
Banys-Paluchowski et al. (189)	Breast reconstruction after mastectomy	Pedicled	–	No
Javeed et al. (192)	Breast reconstruction after mastectomy	Pedicled (fleur-de-lis flap)	–	No
Angrigiani et al. (80)	Breast reconstruction after mastectomy	Pedicled Thoracodorsal Artery Perforator Flap (TDAP)	–	No
Brambilla et al. (169)	Breast reconstruction after mastectomy	Pedicled TDAP (+ implants)	–	No
Homsy et al. (170)	Breast reconstruction after mastectomy	Pedicled and Free TDAP	Internal mammary	No

The LD flap has been extensively used in head and neck reconstruction for a wide spectrum of defects involving the scalp, skull base, orbit, mandible, maxilla, floor of the mouth, tongue, and pharynx. Indications included oncologic resections, trauma, post-burn contractures, infections, and cerebrospinal fluid fistulas. Both pedicled and free flaps were described, although the latter predominated in this region. Recipient vessels commonly included branches of the external carotid system, such as the facial, superficial temporal, superior and inferior thyroid, and occipital arteries. In complex cases, arteriovenous loops or fistulas were used to facilitate microvascular anastomosis. Functional reinnervation was selectively reported, particularly in facial reanimation and tongue reconstruction. These data are detailed in Table 3.

**Table 3:** Use of latissimus dorsi flap for head and neck reconstruction.

Author	Flap indication	Flap type	Recipient vessels	Reinnervation
Sabatier et al. (20)	Craniofacial reconstruction after tumoral excision	Pedicled	–	No
Wilkman et al. (65)	Craniofacial reconstruction after tumoral excision	Pedicled	–	No
Papadopoulos et al. (23)	Craniofacial reconstruction after tumoral excision	Free	Temporal superficial, facial or external carotid	No
Bootz et al. (25)	Anterior cranial reconstruction after tumoral excision	Free	Facial or temporal superficial	No
Schultes et al. (28)	Oral defect and tongue reconstruction after tumoral excision	Free	Facial	Yes (neurorrhaphy to lingual branch of facial nerve)
Kim et al. (44)	Cranial reconstruction after infection or traumatic defect	Free	Temporal superficial	No
Kim et al. (45)	Nasal reconstruction after osteomyelitis	Free	Facial	No
Biglioli et al. (51)	Spheno-orbital reconstruction after tumoral excision	Free (+ rib bone graft)	Temporal superficial or cervical	No
Karle et al. (52)	Jaw reconstruction after tumoral excision	Free chimeric (+ scapular flap + adipocutaneous flap + parascapular flap)	Internal mammary	No
Hillerup et al. (64)	Jaw reconstruction after osteonecrosis	Free (+ iliac bone graft)	Superior thyroid	No
Gilleard et al. (69)	Craniofacial reconstruction after parotid abscess	Free	Facial	Yes (neurorrhaphy to buccal branch of facial nerve)
Longo et al. (76)	Craniofacial reconstruction after tumoral excision	Free	Superior thyroid or facial	No
Singh et al. (78)	Scalp reconstruction after tumoral excision	Free	Temporal superficial	No
Tan et al. (79)	Scalp reconstruction after traumatic defect	Free	A-V loop (facial artery and vein; and small saphenous vein)	No
Mutlu et al. (97)	Scalp reconstruction after tumoral excision	Free	Temporal superficial	No
Kamochi et al. (101)	Orbitomaxillary reconstruction after tumoral excision	Free chimeric (+ scapular flap)	Facial	No
Morice et al. (102)	Scalp reconstruction after traumatic defect	Free	Temporal superficial	No
Park et al. (103)	Facial reconstruction after maxilectomy	Free chimeric (+ scapular flap)	Facial or temporal superficial	No
Tenekeci et al. (104)	Scalp reconstruction after tumoral excision	Free	Temporal superficial	No
Barrientos et al. (119)	Cerebrospinal fluid fistula coverage after tumoral excision and radiation	Free	A-V loop (facial artery and vein; and saphenous vein)	No
Fried et al. (120)	Scalp reconstruction after tumoral excision	Free	Superior thyroid	No
Scaglioni and Giunta (136)	Scalp reconstruction after tumoral excision	Free TDAP	Temporal superficial	No
Wong et al. (121)	Facial reconstruction after arteriovenous malformation	Free (bilateral)	Facial	Yes (neurorrhaphy to zygomatic branch of facial nerve and hypoglossal nerve)
Voss et al. (118)	Jaw reconstruction after tumoral excision	Free chimeric (+ scapular flap)	External carotid	No
Mughal et al. (139)	Scalp reconstruction after tumoral excision	Free	Temporal superficial	No
Xiao et al. (144)	Scalp reconstruction after tumoral excision	Free	Facial	No
Klein et al. (160)	Facial reconstruction after maxillectomy	Free (+ rib bone graft)	Facial	No
Katayama et al. (171)	Jaw reconstruction after tumoral excision	Free	A-V fistula (facial artery and vein)	No
Alessandri-Bonetti et al. (172)	Scalp reconstruction after burn debridement	Pedicled	–	No
Bassiri Gharb et al. (190)	Scalp reconstruction after tumoral excision or cerebrovascular accident	Free (+ rib bone graft)	External carotid, linguofacial trunk or occipital	No
Trapero et al. (193)	Scalp reconstruction after arterio-venous malformation excision	Free chimeric (+ anterior serratus muscle)	Subscapular	No
Thione et al. (183)	Neck and pharyngeal reconstruction after tumoral excision	Free	Inferior thyroid or lingual	No
Jaffar et al. (184)	Head and neck reconstruction after tumoral excision	Free TDAP	Superior thyroid or facial	No
Rodríguez et al. (197)	Mandibular reconstruction after tumoral excision	Pedicled chimeric (+ rib)	–	No

In thoracic and posterior trunk reconstruction, the LD flap was used to address a wide range of defects involving the chest wall, axilla, scapular region and lumbosacral area. Both pedicled and free flaps were employed, either ipsilateral or contralateral to the defect. Multiple technical modifications were reported, including bilobed, reverse-flow, and supercharged designs, as well as chimeric constructs incorporating the serratus anterior, rectus abdominis, pectoralis major, or groin flaps. Pressure ulcer reconstruction of the sacral and ischial regions was also described, predominantly using free LD flaps anastomosed to gluteal vessels. These reconstructions are summarized in Table 4.

**Table 4:** Use of latissimus dorsi flap for thorax and back reconstruction.

Author	Flap indication	Flap type	Recipient vessels	Reinnervation
Amin et al. (88)	Axillary region reconstruction after tumoral excision	Pedicled	–	No
Wormald et al. (66)	Axillary reconstruction after hidradenitis suppurativa	Pedicled TDAP	–	No
La Padula et al. (186)	Axillary reconstruction after hidradenitis suppurativa	Pedicled TDAP	–	No
Chang et al. (154)	Axillary and inguinal reconstruction after burn contracture	Free TDAP	Axillary and femoral	No
Miura et al. (163)	Coverage of aortic prosthesis after omentopexy infection	Free (+ rectus abdominis muscle)	Transverse cervical	No
Bodin et al. (89)	Thoracic wall reconstruction after tumoral excision	Free chimeric (+ anterior serratus muscle)	Contralateral thoracodorsal	No
Lü et al. (96)	Thoracic wall reconstruction after tumoral excision	Pedicled (bilobed)	–	No
Papadakis et al. (111)	Thoracic wall reconstruction after epirubicin extravasation	Pedicled	–	No
Vairinho et al. (112)	Thoracic wall reconstruction after radionecrosis	Pedicled	–	No
Boonipat et al. (128)	Thoracic wall reconstruction after tumoral excision	Pedicled (+ groin flap + rib bone graft)	–	No
Hallock (129)	Thoracic wall reconstruction after thoracotomy wound necrosis	Pedicled (+ serratus anterior muscle)	–	No
Khan et al. (137)	Thoracic wall reconstruction after gunshot	Pedicled	–	No
Fraisse et al. (157)	Thoracic wall reconstruction after epirubicin extravasation	Pedicled	–	No
Zhang et al. (159)	Thoracic wall reconstruction after sternal wound necrosis	Pedicled	–	No
Zhao et al. (158)	Thoracic wall reconstruction after tumoral excision	Pedicled (+ rectus abdominis muscle + pectoralis major muscle)	–	No
Yang et al. (181)	Thoracic wall reconstruction after radionecrosis	Pedicled	–	No
Beltrami et al. (113)	Scapular region reconstruction after tumoral excision	Pedicled (+ scapular prosthesis)	–	No
Kang et al. (114)	Sacral reconstruction after pressure ulcer	Reverse turnover pedicled based on posterior intercostal arteries	–	No
Park and Koh (29)	Ischiosacral reconstruction after pressure ulcer	Free	Superior gluteal	No
Phan et al. (42)	Sacral reconstruction after pressure ulcer	Free (+ serratus anterior muscle)	Inferior gluteal	No
He et al. (43)	Ischial reconstruction after pressure ulcer	Free	Superior gluteal	No
Chang et al. (155)	Sacral reconstruction after pressure ulcer	Free	Gluteal	No
Cigna et al. (71)	Lumbosacral region reconstruction after tumoral excision	Pedicled (supercharged with inferior gluteal artery)	–	No
Tuncer et al. (70)	Lumbosacral region reconstruction after tumoral excision	Free	A-V loop (thoracodorsal artery and vein; and saphenous vein)	No
Miyamoto et al. (98)	Lumbosacral region reconstruction after tumoral excision	Free (flow-through)	Rectal superior (scapular circumflex and anterior serratus as T segment)	No
Hamada et al. (130)	Lumbosacral region reconstruction after tumoral excision	Reverse pedicled based on lumbar arteries (supercharged with anterior serratus artery)	–	No
Menichini et al. (138)	Lumbosacral region reconstruction after tumoral excision	Reverse pedicled based on anterior serratus artery	–	No
Brunetti et al. (156)	Lumbosacral region reconstruction after tumoral excision	Pedicled chimeric (+ fascio-cutaneous propeller flap)	–	No

The LD flap was less frequently reported for abdominal and pelvic reconstruction but was used for complex abdominal wall and pelvic defects following trauma or extensive oncologic resections, including pelvic exenteration. Free, pedicled, and chimeric flaps were described, often incorporating the serratus anterior muscle. In this subgroup, several reports documented spontaneous or intentional flap neurotization with recovery of voluntary muscle contraction. Details are provided in Table 5.

**Table 5:** Use of latissimus dorsi flap for abdomen and pelvis reconstruction.

Author	Flap indication	Flap type	Recipient vessels	Reinnervation
Fujioka et al. (59)	Abdominal wall and pelvis reconstruction after tumoral excision	Free chimeric (+ anterior serratus muscle)	Deep femoral	No
Hornez et al. (61)	Abdominal wall reconstruction after traumatic explosion	Pedicled	–	No
Bodin et al. (106)	Abdominal wall reconstruction after tumoral excision	Free	Deep inferior epigastric	Yes (spontaneous)
Brunetti et al. (174)	Abdominal wall reconstruction after tumoral excision	Free	Deep inferior epigastric	Yes (spontaneous)
Lellouch et al. (178)	Abdominal wall reconstruction after enterocutaneous fistula	Free	Superficial femoral	No
Ahmed et al. (196)	Abdominal wall repair after massive ventral hernia	Free	Deep inferior epigastric	No

Lower extremity reconstruction with the LD flap encompassed defects from the pelvis to the foot, including the thigh, knee, leg, ankle, and foot. Indications included trauma, tumor resection, infection, chronic ischemia, and limb salvage procedures. Free flaps were predominantly used, either as standalone flaps or in chimeric configurations with scapular or serratus anterior components. To extend pedicle length and optimize reach, the thoracodorsal axis was frequently augmented using circumflex scapular or subscapular branches in flow-through or “T” configurations. Functional reinnervation was selectively reported for restoration of ankle dorsiflexion or posterior compartment function. These applications are detailed in Table 6.

**Table 6:** Use of latissimus dorsi flap for lower limb reconstruction.

Author	Flap indication	Flap type	Recipient vessels	Reinnervation
Allen et al. (24)	Coverage of distal leg traumatic amputation	Free chimeric (+ scapular flap)	Posterior tibial	No
Germann et al. (35)	Dorsum of foot reconstruction after tumoral excision in newborn	Free (+ tendon transfer)	Anterior tibial	No
Hierner et al. (39)	Coverage of knee after arthroplasty	Free	Superficial femoral	No
Hwang et al. (49)	Reconstruction of distal leg after Marjolin ulcer excision	Free (use of pedicle as a T-junction to facilitate fibular free flap)	Anterior tibial	No
Nazerani et al. (48)	Reconstruction of knee after traumatic defect	Free (+ rib bone graft)	Popliteal	No
Patel et al. (55)	Coverage of distal leg traumatic amputation and extensor muscle resection	Free chimeric (+ anterior serratus muscle + adipocutaneous flap)	Anterior tibial	Yes (neurorrhaphy to deep peroneal nerve)
Venkatramani et al. (50)	Reconstruction of knee after traumatic defect	Free	Descending genicular	No
Jain et al. (73)	Reconstruction of knee after traumatic defect	Free	Anterior tibial	No
Miyamoto et al. (77)	Reconstruction of distal leg after tumoral excision	Free (flow-through)	Anterior tibial and posterior tibial (scapular circumflex as T segment)	No
Gao et al. (93)	Reconstruction of distal leg after traumatic defect	Free (flow-through)	Posterior tibial (scapular circumflex and subscapular as T segment)	No
Gencel et al. (94)	Reconstruction of distal leg after electrical burn debridement	Free (cross flow-through)	Contralateral posterior tibial (scapular circumflex, subscapular and anterior serratus as T segment)	No
Hwang et al. (95)	Reconstruction of leg after tibial open fracture	Free (+ iliac bone graft)	Anterior tibial or posterior tibial	No
Rednam and Rinker (83)	Reconstruction of leg after tibial open fracture	Free	Anterior tibial	Yes (neurorrhaphy to motor branch of soleus nerve)
Tashiro et al. (122)	External hemipelvectomy reconstruction after tumoral excision	Free (flow-through)	Deep inferior epigastric (scapular circumflex as T segment)	Yes (spontaneous)
Chim et al. (8)	Leg, ankle and foot reconstruction after traumatic defect	Free	Anterior tibial or posterior tibial	No
Dragos et al. (140)	Reconstruction of knee after traumatic defect	Free (anterior serratus muscle (+ rib bone graft)	Posterior tibial	No
Münchow et al. (149)	Coverage of knee after infected arthroplasty	Free	A-V loop (common femoral artery and vein; and contralateral great saphenous vein)	No
Raymond et al. (150)	Coverage of knee after arthroplasty	Free	Superficial femoral	No
Amendola et al. (161)	Reconstruction of leg and foot after traumatic defect	Free chimeric (+ scapular flap + groin flap)	Descending branch of lateral femoral circumflex and anterior tibial	No
He et al. (162)	Reconstruction of knee, leg, ankle and foot after traumatic defect or tumoral excision	Free	Descending branch of lateral femoral circumflex, descending genicular, lateral superior genicular, popliteal, anterior tibial, posterior tibial, medial sural, peroneal	No
Kagaya et al. (173)	Coverage of Lisfranc stump in patients with chronic ischemia	Free	Arterialized vein (superficial femoral artery bypass to great saphenous vein)	No
Sui et al. (185)	Leg, knee and foot reconstruction after trauma, tumoral excision and burn scar	Free TDAP (+ latissimus dorsi muscle)	Medial gluteal, superficial femoral, posterior tibial or anterior tibial	No
Fragomen et al. (200)	Lower limb reconstruction and lymphedema treatment	Free chimeric (+ fasciocutaneous flap + lymph node transfer)	Posterior tibial	No

Upper extremity reconstruction included defects of the shoulder, arm, elbow, forearm, hand, and digits. Both pedicled and free LD flaps were reported, occasionally combined with bone grafts for skeletal reconstruction. Recipient vessels most commonly included the brachial and radial arterial systems. In selected cases, targeted muscle reinnervation using median, ulnar, or radial nerves was described, particularly in reconstructions aiming to restore elbow or arm function and to reduce neuropathic pain. These data are summarized in Table 7.

**Table 7:** Use of latissimus dorsi flap for upper limb reconstruction.

Author	Flap indication	Flap type	Recipient vessels	Reinnervation
Meiners et al. (33)	Shoulder reconstruction after infection of neuropathic shoulder	Pedicled	–	No
Ozer et al. (41)	Elbow reconstruction after open fracture and loss of soft tissue	Pedicled (+ rib bone graft)	–	No
Qu et al. (47)	Elbow and arm reconstruction after traumatic defects	Pedicled	–	No
Ng et al. (87)	Elbow reconstruction after tumoral excision	Free	Brachial	No
Kim et al. (62)	Multiple digit reconstruction after traumatic defects or infection	Free (+ iliac bone grafts)	Radial y dorsal radial	No
Kesiktas et al. (75)	Coverage of transhumeral stump after electric burn debridement	Pedicled	–	No
Cadenelli et al. (90)	Shoulder and arm reconstruction after tumoral excision	Pedicled	–	No
Anghel et al. (179)	Coverage of high transhumeral stump after high-energy trauma	Pedicled	–	Yes (neurorrhaphy to ulnar and median nerve)
Olvera-Yarza et al. (180)	Coverage of axillary region after hidradenitis suppurativa debridement	Pedicled	–	No
Marchesi et al. (116)	Coverage of axillary region after hidradenitis suppurativa debridement	Pedicled	–	No

Tables 8, 9 specifically summarize functional reconstructions using innervated and non-innervated LD flaps, respectively. Innervated flaps were most frequently employed for facial reanimation, urologic indications such as phalloplasty (in gender affirmation surgery as well as traumatic and congenital amputations) and detrusor myoplasty, upper and lower limb motor restoration and diaphragmatic reconstruction. Non-innervated flaps were primarily used when passive functional support or dynamic stabilization was sufficient, such as in diaphragmatic, shoulder, elbow, and pharyngeal reconstruction.

**Table 8:** Use of innervated latissimus dorsi flap for functional reconstruction.

Author	Flap indication	Flap type	Recipient vessels	Reinnervation
Perovic et al. (38)	Phalloplasty in congenital aphallia, iatrogenic and traumatic amputations	Free	Femoral	Yes (neurorrhaphy to ilioinguinal nerve)
Adamyan et al. (177)	Phalloplasty in gender affirming surgery (female to male)	Free	Deep epigastric	Yes (neurorrhaphy to motor branch of obturator nerve)
Djordjevic et al. (123)	Phalloplasty in gender affirming surgery (female to male)	Free	Femoral	Yes (neurorrhaphy to ilioinguinal nerve)
Djordjevic et al. (37)	Phalloplasty in congenital aphallia	Free	Femoral	Yes (neurorrhaphy to ilioinguinal nerve)
Gakis et al. (40)	Functional detrusor myoplasty in patients with bladder acontractility caused by lower motor neuron lesion	Free	Inferior epigastric	Yes (neurorrhaphy to 12th intercostal nerve)
Rednam and Rinker (83)	Functional reconstruction of posterior compartment of leg after traumatic defect	Free	Tibioperoneal trunk	Yes (neurorrhaphy to soleus branch of tibial nerve)
Patel et al. (55)	Restoration of ankle dorsiflexion after traumatic defect	Free	Anterior tibial	Yes (neurorrhaphy to deep peroneal nerve)
Anghel et al. (179)	Restoration of flexion and extension of arm after high transhumeral amputation	Pedicled	–	Yes (neurorrhaphy to radial nerve and median nerve)
Fujioka and Koga (191)	Functional reconstruction of trapezius muscle after tumoral excision	Pedicled	–	Yes (neurorrhaphy to accessory nerve)
Nicoli et al. (82)	Functional triceps reconstruction after tumoral excision	Free (supercharged) (+ groin flap + lymphatic nodes transfer + sural nerve graft)	Radial	Yes (neurorrhaphy to cubital nerve)
Trimaille et al. (56)	Restoration of wrist and finger flexion and extension after congenital Volkmann contracture	Free	Radial	Yes (neurorrhaphy to median nerve)
Horta et al. (67)	Phrenic nerve neurotization for restoring physiological motion in congenital diaphragmatic hernia	Pedicled	-	Yes (neurorrhaphy to phrenic nerve)
Gilleard et al. (69)	Craniofacial reconstruction after parotid abscess	Free	Facial	Yes (neurorrhaphy to buccal branch of facial nerve)
Takushima et al.	Facial reanimation after tumor excision, Bell's palsy, trauma or stroke	Free	Facial	Yes (neurorrhaphy to buccal branch of contralateral facial nerve)
Okazaki et al. (68)	Facial reanimation after tumoral excision or congenital cause	Free (bilobed) (+ fascia lata graft)	Facial	Yes (neurorrhaphy to buccal branch of facial nerve; and masseteric branch of mandibular nerve)
Homma et al. (99)	Facial reanimation after tumoral excision	Free (bilobed) (+ fascia lata graft)	Facial	Yes (neurorrhaphy to masseteric branch of mandibular nerve)
Matsumine et al. (134)	Facial reanimation after tumoral excision or traumatic defect	Free chimeric (+ anterior serratus muscle)	Facial	Yes (neurorrhaphy to buccal branch of facial nerve and masseteric branch of mandibular nerve)
Wong et al. (121)	Facial reanimation after arteriovenous malformation excision	Free	Facial	Yes (neurorrhaphy to zygomatic branch of facial nerve)
Ogawa et al. (147)	Facial reanimation after tumoral excision	Free chimeric (+fascio-adipo-cutaneous flap + fascia lata graft)	Facial	Yes (neurorrhaphy to buccal branch and zygomatic branch of facial nerve)
Yoo et al. (165)	Facial reanimation after tumoral excision or traumatic defect	Free	Facial	Yes (neurorrhaphy to masseteric branch of trigeminal nerve and facial nerve)
Bedarida et al. (146)	Facial reanimation after tumoral excision or trauma	Free TDAP	External carotid	Yes (neurorrhaphy to trunk and distal branches of facial nerve)
Panzenbeck et al. (199)	Facial reanimation after congenital Moebius syndrome	Free (bilateral)	Facial	Yes (neurorrhaphy to masseteric, hypoglossal or accessory nerves)
Ogawa et al. (148)	Oral depressor muscles reinnervation after tumoral excision	Free chimeric (+ fascio-adipo-cutaneous flap)	Facial and superior thyroid	Yes (neurorrhaphy to marginal branch and buccal branch of facial nerve)
Wu et al. (194)	Functional tongue reconstruction after tumoral excision	Free TDAP	Facial	Yes (neurorrhaphy to hypoglossal nerve)
Mordovskiy et al. (198)	Functional tongue and suprahyoid muscles reconstruction after tumoral excision	Free chimeric (+ serratus anterior muscle)	*Unknown*	Yes (neurorrhaphy to hypoglossal nerve)

**Table 9:** Use of non-innervated latissimus dorsi flap for functional reconstruction.

Author	Flap indication	Flap type	Recipient vessels	Reinnervation
Marque et al. (187)	Phalloplasty in gender affirming surgery (female to male)	TDAP	Inferior epigastric	No
Nasır (195)	Phalloplasty in gender affirming surgery (female to male), congenital aphallia and traumatic amputations	Free	Superior femoral or common femoral	No
Gangurde et al. (46)	Thoracic wall reconstruction after Jarcho-Levin syndrome	Pedicled	–	No
Tsai et al. (53)	Bronchopleural fistula collapse	Pedicled	–	No
Zimmermann et al. (21)	Closure of acute bronchial stump after pneumonectomy	Pedicled	–	No
Yap et al. (22)	Repair of thoracic oesophageal perforation	Pedicled	–	No
Bianchi et al. (19)	Diaphragmatic hernia restoration in newborns	Reverse pedicled	–	No
Wallace and Roden (26)	Diaphragmatic hernia restoration in newborns	Reverse pedicled	–	No
Whetzel et al. (27)	Diaphragmatic hernia restoration in kids	Reverse pedicled	–	No
Bedini et al. (30)	Diaphragmatic reconstruction after pneumonectomy	Reversed pedicled	–	No
McConkey et al. (36)	Diaphragmatic reconstruction after tumoral excision	Pedicled	–	No
Buchanan et al. (84)	Functional pectoral muscle reconstruction in Poland syndrome	Pedicled	–	No
Muramatsu et al. (60)	Functional deltoid muscle reconstruction after tumoral excision	Pedicled	–	No
De Smet et al. (34)	Functional deltoid muscle reconstruction after irreparable axillary lesion	Pedicled	–	No
Bota et al. (175)	Functional deltoid muscle reconstruction after trauma	Pedicled	–	No
Ortmaier et al. (107)	Mobility restoration of rotator cuff in combination with humeral arthroplasty	Pedicled	–	No
Valenti et al. (153)	Mobility restoration of rotator cuff in combination with humeral arthroplasty	Pedicled	–	No
Alonso-Rodriguez Piedra et al. (176)	Mobility restoration of rotator cuff in combination with humeral arthroplasty	Pedicled	–	No
Kim et al. (182)	Reconstruction of chronic seroma pocket in axillary fossa	Pedicled	–	No
Vibhakar et al. (57)	Breast reconstruction and lymphedema treatment	Pedicled (bilateral) (+ lateral thoracic nodes transfer)	–	No
Azab and Alsabbahi (100)	Restoration of elbow flexion after traumatic defect	Pedicled	–	No
Ozaniak et al. (166)	Restoration of elbow flexion after tumoral excision	Pedicled	–	No
Alshammari et al. (152)	Restoration of elbow flexion after traumatic defect	Pedicled	–	No
Ihara et al. (31)	Restoration of flexion and extension of fingers after traumatic defects	Pedicled	–	Yes (spontaneous)
Takahashi et al. (105)	Restoration of finger extension after traumatic defects	Pedicled	–	Yes (spontaneous)
Ozcan Akcal et al. (91)	Functional foot reconstruction after traumatic defects	Free chimeric (+ scapular flap)	Anterior tibial and posterior tibial	No
Ju et al. (115)	Chronic osteomyelitis treatment in lower limb	Free (+ iliac bone graft)	Anterior tibial and posterior tibial	No
Fragomen et al. (200)	Lower limb reconstruction and lymphedema treatment	Free chimeric (+ fasciocutaneous flap + lymph node transfer)	Posterior tibial	No
Biglioli et al. (51)	Spheno-orbital reconstruction after tumoral excision	Free (+ rib bone graft)	Temporal superficial or cervical	No
Park et al. (103)	Maxillary reconstruction after tumoral excision	Free chimeric (+ scapular flap)	Facial or temporal superficial	No
Kamochi et al. (101)	Orbitomaxillary reconstruction after tumoral excision	Free chimeric (+ scapular flap)	Facial	No
Özkan et al. (135)	Functional lower lip reconstruction after tumoral excision of traumatic defect	Free	Facial or superior thyroid	Yes (spontaneous)
Thione et al. (183)	Pharyngeal reconstruction after tumoral excision	Free	Inferior thyroid or lingual	No
Bach et al. (81)	Oral and pharyngeal reconstruction after tumoral excision	Free TDAP	Thyroid or facial	No
Rodríguez et al. (197)	Mandibular reconstruction after tumoral excision	Pedicled chimeric (+ rib)	–	No

## Discussion

The latissimus dorsi flap remains a a cornerstone of reconstructive surgery owing to its reliable vascular anatomy, long and adaptable pedicle, and broad arc of rotation. These characteristics allow the flap to be harvested as pedicled or free and to be configured in chimeric, bilobed, reverse-flow, or supercharged designs, thereby substantially expanding its reconstructive versatility.

Across the literature, the LD flap has demonstrated value not only as a robust coverage option but also as a powerful tool for functional reconstruction, in both innervated and non-innervated forms, depending on the clinical objective. Nevertheless, despite this broad applicability, its use consistently follows recognizable indication-based patterns related to flap design and reconstructive goals.

As a general concept, pedicled flaps are predominantly described for defects located within their arc of rotation, most commonly involving the breast, chest wall, back, shoulder, and proximal upper extremity. In contrast, free flaps are more frequently reported for distant, extensive, or complex defects, including head and neck reconstruction, distal upper and lower extremity defects, abdominal wall and pelvic reconstruction.

Innervated, often bilobed or chimeric, LD flaps are primarily indicated when active functional restoration is required. Across multiple anatomical regions, targeted neurorrhaphy has enabled recovery of voluntary muscle contraction, thereby extending the role of the LD flap well beyond static coverage. Dynamic facial reanimation emerges as the most frequently reported functional indication, reflecting the favourable muscle excursion of the muscle and its compatibility with facial, masseteric, and hypoglossal nerve branches. In the upper extremity, innervated flaps have been successfully applied for reconstruction of major functional units, including elbow extension, forearm flexion and extension, and targeted muscle reinnervation following amputation, demonstrating the adaptability of the flap in restoring motor control and reducing neuropathic pain. Similarly, in the lower extremity, reinnervated flaps have contributed to the restoration of ankle and foot function following traumatic or oncologic defects. Beyond musculoskeletal reconstruction, innervated flaps have been described in visceral and dynamic reconstructions, such as detrusor myoplasty, neo-phalloplasty, diaphragmatic and pharyngeal reconstruction, and abdominal wall restoration.

By contrast, non-innervated LD flaps remain highly relevant when passive functional support or durable structural restoration is sufficient. Their predominant use as pedicled flaps in breast and thoracic reconstruction and as free flaps in craniofacial reconstruction reflects their reliability and acceptable outcomes.

Lastly, muscle-sparing and perforator-based flaps, including TDAP, are preferentially described in situations where minimization of donor-site morbidity is a primary concern, particularly in breast and trunk reconstruction. Their use reflects an evolving reconstructive philosophy that prioritizes functional preservation while maintaining reliable soft-tissue coverage.

Tables 10, 11 are organized according to flap transfer modality, distinguishing between pedicled and free latissimus dorsi flaps, and further stratified by innervated and non-innervated designs within each category. This structure provides a clear and practical overview of the reported indications, allowing surgeons to readily identify reconstructive strategies that have been described for specific anatomical regions and functional objectives. This framework is intended as a descriptive guide based on the existing literature, rather than a prescriptive algorithm, and aims to support indication-driven decision-making rather than subjective preference.

**Table 10:** Summary of All A applications of the free Latissimus dorsi flap.

Location	Coverage	Functional non-innervated	Functional innervated
Breast	Breast reconstruction (internal mammary vessels as receptors)		–
Head and neck	Coverage of skull base, scalp and craniofacial region following tumor resection, infection, trauma or osteonecrosis	Functional pharyngeal and lower lip reconstruction following trauma or tumor	Functional reconstruction of floor of mouth, tongue and cervicofacial region following tumor resection and infection Dynamic facial reanimation
Thorax and back	Coverage of chest wall, axilla, scapula, lumbosacral or ischiosacral region following tumor resection, pressure ulcer or infection	Coverage of aortic prosthesis following infection and extended thoracic wall and lumbosacral region	–
Abdomen and pelvis	Coverage of abdominal wall following tumor resection, ventral hernias or entero-cutaneous fistulas	Functional abdominal wall reconstruction following tumor resection or trauma	Neo-phalloplasty (gender affirmation surgery or traumatic and congenital amputations) Detrusor reinnervation following neuronal lesion
Lower limb	Coverage of thigh, knee, leg and foot following trauma, infection, tumor resection, chronic or burns	Functional stump, knee and foot reconstruction following trauma, chronic osteomyelitis and tumor resection Lymphedema treatment	Restoration of ankle dorsiflexion and posterior compartment function following traumatic amputations
Upper limb	Coverage of arm, elbow and fingers following tumor resection, trauma or infection	Functional elbow and hand reconstruction with skeletal components	Restoration of elbow flexion, wrist flexion and extension following tumor resection and Volkmann's contracture Targeted muscle reinnervation

**Table 11:** Summary of All applications of the pedicled Latissimus dorsi flap.

Location	Coverage	Functional non-innervated	Functional innervated
Breast	Breast reconstruction after mastectomy or lumpectomy; volume replacement combined with implants, expanders or fat grafting		–
Head and neck	Coverage of scalp and craniofacial region following tumoral resection or burns	Maxilla reconstruction following tumoral excision	–
Thorax and back	Coverage of chest wall, axilla, scapula and posterior trunk following tumoral resection, or tissue necrosis (secondary to extravasation, radionecrosis, pressure sores)	Repair of congenital diaphragmatic hernia or post-oncologic defects, closure of bronchopleural fistulae, repair of esophageal perforation and chest reconstruction in Poland and Jarcho-Levin syndromes	Diaphragm reinnervation following congenital diaphragmatic hernia and reinnervation of shoulder after tumoral resection
Abdomen and pelvis	Coverage of abdomen and pelvis following trauma	–	–
Lower limb	–	–	–
Upper limb	Coverage of shoulder, arm and elbow following trauma, burns, infection, hidradenitis or tumoral resection	Functional repair of shoulder and upper limb following tumor or trauma Lymphedema or axillary lymphorrhea treatment	

Several limitations of this qualitative systematic review must be acknowledged. The available evidence is largely derived from case reports and case series, with significant heterogeneity in indications, flap design, nerve selection, and outcome assessment. Objective functional metrics and long-term follow-up are inconsistently reported, precluding quantitative comparison or meta-analysis. Future studies specifically designed to compare reconstructive strategies—ideally through well-structured cohort studies or dedicated systematic reviews—would be valuable to further define optimal flap selection across different clinical scenarios. Nevertheless, the consistency of reliable reconstructive and functional outcomes across a wide range of anatomical regions supports the continued clinical relevance and adaptability of the LD flap in contemporary reconstructive surgery.

## Conclusion

In conclusion, the latissimus dorsi flap remains a cornerstone of reconstructive surgery, demonstrating versatility across multiple anatomical regions. Whether used for oncologic, traumatic, or congenital defect reconstruction, it can be employed in multiple configurations, pedicled or free, reinnervated or not, attaining optimal functional and aesthetic outcomes. Its applications continue to evolve, with advancements in microsurgical techniques and functional restoration enhancing its role in complex defect reconstruction.

Our work is the first of its kind to describe and compile all the reported applications of the latissimus dorsi flap published in literature to date, serving as a reference for surgeons to review whenever considering whether the latissimus dorsi flap might be a possible solution for any defect they may be confronted with.

## Data Availability

The original contributions presented in the study are included in the article/Supplementary Material, further inquiries can be directed to the corresponding author.
