# Supplementary material for: Latissimus dorsi flap: a comprehensive systematic review of traditional and novel applications
Source: Front Surg. 2026 Mar 13;13:1752461. doi: 10.3389/fsurg.2026.1752461 (PMC13021856; doi:10.3389/fsurg.2026.1752461)
Supplement: Supplementary file 1 [file Table1.docx]

**SUPPLEMENTARY MATERIAL**

Supplementary Table 1: Results of the quality assessment (Joanna Briggs Institute (JBI) Critical Appraisal Checklist for Case Reports) for each included study.

| AUTHOR | TYPE OF STUDY | OVERALL APPRAISAL | QUALITY |
| --- | --- | --- | --- |
| Bianchi et al.[19] | Case series | Include | High |
| Sabatier et al.[20] | Case series | Include | High |
| Zimmermann et al.[21] | Case report | Include | High |
| Yap et al.[22] | Case report | Include | High |
| Papadopoulos et al.[23] | Case series | Include | High |
| Allen et al.[24] | Case series | Include | High |
| Bootz et al.[25] | Case series | Include | High |
| Wallace and Roden[26] | Case series | Include | High |
| Whetzel et al.[27] | Case report | Include | High |
| Schultes et al.[28] | Case report | Include | High |
| Park and Koh[29] | Case report | Include | High |
| Bedini et al.[30] | Case series | Include | High |
| Ihara et al.[31] | Case series | Include | High |
| Dixon et al.[32] | Case series | Include | High |
| Meiners[33] | Case report | Include | High |
| De Smet et al.[34] | Case report | Include | High |
| Germann et al.[35] | Case report | Include | High |
| McConkey et al.[36] | Case report | Include | High |
| Djordjevic et al.[37] | Case series | Include | High |
| Perovic et al.[38] | Case series | Include | High |
| Hierner et al.[39] | Case series | Include | High |
| Gakis et al.[40] | Case series | Include | High |
| Ozer et al.[41] | Case report | Include | High |
| Phan et al.[42] | Case series | Include | High |
| He et al.[43] | Case series | Include | High |
| Kim et al.[44] | Case series | Include | High |
| Kim et al.[45] | Case report | Include | High |
| Gangurde et al.[46] | Case series | Include | High |
| Qu et al.[47] | Case series | Include | High |
| Nazerani et al.[48] | Case series | Include | High |
| Hwang et al.[49] | Case report | Include | High |
| Venkatramani et al.[50] | Case series | Include | High |
| Biglioli et al.[51] | Case series | Include | High |
| Karle et al.[52] | Case series | Include | High |
| Tsai et al.[53] | Case report | Include | High |
| Takushima et al.[54] | Case series | Include | High |
| Patel et al.[55] | Case report | Include | High |
| Trimaille et al.[56] | Case report | Include | High |
| Vibhakar et al.[57] | Case report | Include | High |
| Endara et al.[58] | Case report | Include | High |
| Fujioka et al.[59] | Case report | Include | High |
| Muramatsu et al.[60] | Case series | Include | High |
| Hornez et al.[61] | Case report | Include | High |
| Kim et al.[62] | Case report | Include | High |
| Santanelli di Pompeo et al.[63] | Case series | Include | High |
| Hillerup et al.[64] | Case series | Include | High |
| Wilkman et al.[65] | Case series | Include | High |
| Wormald et al.[66] | Case series | Include | High |
| Horta et al.[67] | Case report | Include | High |
| Okazaki et al.[68] | Case series | Include | High |
| Gilleard et al.[69] | Case report | Include | High |
| Tuncer et al.[70] | Case report | Include | High |
| Cigna et al.[71] | Case report | Include | High |
| Højvig y Bonde[72] | Case series | Include | High |
| Jain et al.[73] | Case report | Include | High |
| Kaur et al.[74] | Case series | Include | High |
| Kesiktas et al.[75] | Case series | Include | High |
| Longo et al.[76] | Case series | Include | High |
| Miyamoto et al.[77] | Case series | Include | High |
| Singh et al.[78] | Case series | Include | High |
| Tan et al.[79] | Case series | Include | High |
| Angrigiani et al.[80] | Case series | Include | High |
| Bach et al.[81] | Case series | Include | High |
| Nicoli et al.[82] | Case report | Include | High |
| Rednam and Rinker[83] | Case report | Include | High |
| Buchanan et al.[84] | Case report | Include | High |
| Banys-Paluchowski et al.[85] | Case report | Include | High |
| He et al.[86] | Case series | Include | High |
| Ng et al.[87] | Case report | Include | High |
| Amin et al.[88] | Case report | Include | High |
| Bodin et al.[89] | Case report | Include | High |
| Cadenelli et al.[90] | Case report | Include | High |
| Ozcan Akcal et al.[91] | Case series | Include | High |
| Correia Anacleto et al.[92] | Case series | Include | High |
| Gao et al.[93] | Case series | Include | High |
| Gencel et al.[94] | Case series | Include | High |
| Hwang et al.[95] | Case series | Include | High |
| Lü et al.[96] | Case series | Include | High |
| Mutlu et al.[97] | Case report | Include | High |
| Miyamoto et al.[98] | Case series | Include | High |
| Homma et al.[99] | Case report | Include | High |
| Azab and Alsabbahi[100] | Case series | Include | High |
| Kamochi et al.[101] | Case series | Include | High |
| Morice et al.[102] | Case series | Include | High |
| Park et al.[103] | Case series | Include | High |
| Tenekeci et al.[104] | Case report | Include | High |
| Takahashi et al.[105] | Case report | Include | High |
| Bodin et al.[106] | Case series | Include | High |
| Ortmaier et al.[107] | Case series | Include | High |
| Cook et al.[7] | Case series | Include | High |
| De Runz et al.[108] | Case series | Include | High |
| DeLong et al.[109] | Case series | Include | High |
| Yuan et al.[110] | Case series | Include | High |
| Papadakis et al.[111] | Case report | Include | High |
| Vairinho et al.[112] | Case report | Include | High |
| Beltrami et al.[113] | Case series | Include | High |
| Kang et al.[114] | Case series | Include | High |
| Ju et al.[115] | Case series | Include | High |
| Marchesi et al.[116] | Case series | Include | High |
| Lai et al.[117] | Case report | Include | High |
| Voss et al.[118] | Case series | Include | High |
| Barrientos et al.[119] | Case report | Include | High |
| Fried et al.[120] | Case report | Include | High |
| Wong et al.[121] | Case report | Include | High |
| Tashiro et al.[122] | Case series | Include | High |
| Djordjevic et al.[123] | Case series | Include | High |
| Dutra et al.[124] | Case series | Include | High |
| Maselli et al.[125] | Case series | Include | High |
| Mericli et al.[126] | Case series | Include | High |
| Motono et al.[127] | Case report | Include | High |
| Boonipat et al.[128] | Case report | Include | High |
| Hallock[129] | Case report | Include | High |
| Hamada et al.[130] | Case report | Include | High |
| Li et al.[131] | Case series | Include | High |
| Liu et al.[132] | Case series | Include | High |
| Lohana et al.[133] | Case series | Include | High |
| Matsumine et al.[134] | Case series | Include | High |
| Özkan et al.[135] | Case series | Include | High |
| Scaglioni and Giunta[136] | Case series | Include | High |
| Khan et al.[137] | Case report | Include | High |
| Menichini et al.[138] | Case report | Include | High |
| Mughal et al.[139] | Case series | Include | High |
| Chim et al.[8] | Case series | Include | High |
| Dragos et al.[140] | Case report | Include | High |
| Cha et al.[15] | Case series | Include | High |
| De Lorenzi et al.[141] | Case series | Include | High |
| Piat et al.[142] | Case series | Include | High |
| Sakharpe et al.[143] | Case report | Include | High |
| Xiao et al.[144] | Case report | Include | High |
| Song et al.[145] | Case series | Include | High |
| Bedarida et al.[146] | Case series | Include | High |
| Ogawa et al.[147] | Case report | Include | High |
| Ogawa et al.[148] | Case series | Include | High |
| Münchow et al.[149] | Case report | Include | High |
| Raymond et al.[150] | Case series | Include | High |
| Ahmed et al.[17] | Case series | Include | High |
| Casella et al.[151] | Case series | Include | High |
| Couto-González et al.[16] | Case series | Include | High |
| Alshammari et al.[152] | Case report | Include | High |
| Valenti et al.[153] | Case series | Include | High |
| Chang et al.[154] | Case series | Include | High |
| Chang et al.[155] | Case series | Include | High |
| Brunetti et al.[156] | Case report | Include | High |
| Fraisse et al.[157] | Case report | Include | High |
| Zhao et al.[158] | Case report | Include | High |
| Zhang et al.[159] | Case series | Include | High |
| Klein et al.[160] | Case report | Include | High |
| Amendola et al.[161] | Case report | Include | High |
| He et al.[162] | Case series | Include | High |
| Miura et al.[163] | Case report | Include | High |
| Feng et al.[164] | Case series | Include | High |
| Yoo et al.[165] | Case series | Include | High |
| Ozaniak et al.[166] | Case report | Include | High |
| Zheng et al.[167] | Case series | Include | High |
| Lee et al.[168] | Case series | Include | High |
| Brambilla et al.[169] | Case series | Include | High |
| Homsy et al.[170] | Case series | Include | High |
| Katayama et al.[171] | Case series | Include | High |
| Alessandri-Bonetti et al.[172] | Case report | Include | High |
| Kagaya et al.[173] | Case report | Include | High |
| Brunetti et al.[174] | Case report | Include | High |
| Chen et al.[9] | Case series | Include | High |
| Bota et al.[175] | Case report | Include | High |
| Alonso-Rodriguez Piedra et al.[176] | Case series | Include | High |
| Adamyan et al.[177] | Case series | Include | High |
| Lellouch et al.[178] | Case report | Include | High |
| Anghel et al.[179] | Case report | Include | High |
| Olvera-Yarza et al.[180] | Case report | Include | High |
| Yang et al.[181] | Case report | Include | High |
| Kim et al.[182] | Case report | Include | High |
| Thione et al.[183] | Case series | Include | High |
| Jaffar et al.[184] | Case series | Include | High |
| Sui et al.[185] | Case series | Include | High |
| La Padula et al.[186] | Case series | Include | High |
| Marque et al.[187] | Case report | Include | High |
| D’Alessandro et al.[188] | Case series | Include | High |
| Banys-Paluchowski et al.[189] | Case series | Include | High |
| Bassiri Gharb et al.[190] | Case series | Include | High |
| Fujioka and Koga[191] | Case report | Include | High |
| Javeed et al.[192] | Case series | Include | High |
| Trapero et al.[193] | Case report | Include | High |
| Wu et al.[194] | Case series | Include | High |
| Nasır[195] | Case series | Include | High |
| Ahmed et al.[196] | Case series | Include | High |
| Rodríguez et al.[197] | Case report | Include | High |
| Mordovskiy et al.[198] | Case series | Include | High |
| Panzenbeck et al.[199] | Case series | Include | High |
| Fragomen et al.[200] | Case report | Include | High |
